# MYC target gene activation in chronic lymphocytic leukemia and richter transformation: links to aggressiveness and tumor microenvironment interactions

**DOI:** 10.3389/fphar.2025.1642458

**Published:** 2025-08-15

**Authors:** M. Tsagiopoulou, S. Rashmi, M. Chatziaslani, I. Gut

**Affiliations:** ^1^ Centro Nacional de Analisis Genomico (CNAG), Barcelona, Spain; ^2^ Aristotle University of Thessaloniki (AUTh), Thessaloniki, Greece; ^3^ International Hellenic University, Thessaloniki, Greece; ^4^ Universitat de Barcelona (UB), Barcelona, Spain

**Keywords:** CLL, MYC, single cell RNAseq, bulk RNAseq, richter transformation

## Abstract

Chronic Lymphocytic Leukemia (CLL) is characterized by clinical and biological heterogeneity, with a subset of patients progressing to Richter Transformation (RT), an aggressive lymphoma. This study explores MYC target gene activation across various CLL stages and disease subgroups using bulk RNAseq and single-cell RNAseq data. Our findings reveal increased MYC activation in unmutated IGHV CLLs, trisomy 12 cases, and RT stages. In RT, MYC activation is independent of B-cell receptor signaling, correlating instead with cell cycling and TLR9 interactions, indicating alternative survival mechanisms. High MYC activation correlates with shorter time to first treatment and enhances tumor microenvironment interactions, particularly with myeloid cells. These results underscore MYC’s significant role in CLL progression and RT, supporting MYC’s potential as a target for stratifying CLL patients and developing therapeutic strategies.

## 1 Introduction

Chronic Lymphocytic Leukemia (CLL) is a mature B cell malignancy noted for its clinical and biological heterogeneity (Delgado et al., 2020). This variability stems from a complex interaction between genetic factors, epigenetic modification, and the tumor microenvironment (TME). A subset of patients undergoing treatment may experience transformation into an aggressive form of lymphoma, a progression known as Richter Transformation (RT) (Wang and Ding, 2020). Key drivers of RT involve alterations in critical cellular pathways including the cell cycle, *MYC*, *NOTCH* and *NF-κB* pathways (Nadeu et al., 2022). Transcriptomic analyses have revealed that RT cells exhibit activation of the oxidative phosphorylation pathway alongside a downregulation of B-cell receptor (BCR) signaling (Nadeu et al., 2022). In CLL, activation of the BCR is known to upregulate *MYC* expression via a BTK-dependent mechanism (Yeomans et al., 2016). Increased *MYC* target gene expression was found in lymph nodes with corresponding increases in *MYC* protein levels in both lymph nodes and unmutated CLL (U-CLL) (Herishanu et al., 2011). While genetic events in *MYC* are rare in CLL (Huh et al., 2008), the transcriptomic activation of *MYC* remains unexplored. Therefore, this study aims to explore the activation of *MYC* target genes across various stages of CLL—including diagnosis, progression, relapse, and RT and various disease subgroups (*e.g.*, unmutated IGHV-CLL cases (U-CLLs), genetic alterations).

## 2 Materials and methods

### 2.1 Study group

We used a comprehensive dataset comprising 263 bulk RNAseq CLL samples from the ICGC cohort (Knisbacher et al., 2022; https://www.cllmap.org/downloads.html). None of the cases carried MYC genetic alterations. Considering the clinical trajectory of CLL, 12 bulk RNAseq samples from 6 CLL patients pre- and post-RT (Nadeu et al., 2022) were included.

At single-cell resolution, we analyzed single-cell RNAseq data from 40,725 cells, including CLL, TME, and RT (Nadeu et al., 2022). The bulk and single-cell data from the Nadeu et al. (2022), study included case 3,299, which carried a MYC missense mutation in both the CLL and RT phases, and case 365, which had a subclonal translocation in the CLL phase that became clonal in the RT phase. For further validation, we also used data from 13,280 cells before ibrutinib treatment (comprising CLL and TME) in four patients, as well as 10,299 CLL cells before ibrutinib treatment and 9,450 CLL cells during ibrutinib treatment at three time points (Rendeiro et al., 2020).

### 2.2 Bulk RNAseq analysis

For the ICGC data, we used TPM (transcripts per million) values, a normalization method that explicitly corrects for both sequencing depth and gene length, thereby minimizing gene length bias when calculating sample-level scores. Moreover, batch effect correction using Combat was performed, considering the number of counts per sample as a co-factor for the ICGC cohort. Differential expression was examined using limma. For the RT cohort, we processed RNAseq data using the tximport package in R to import transcript-level quantifications from Kallisto. The imported data were then used to create a DESeq2 dataset, with sample metadata including case and diagnosis information, and differential expression analysis was performed to identify significant changes between conditions. Finally, variance stabilizing transformation (VST) was applied to the dataset, and the resulting transformed expression values were used for downstream analyses.

### 2.3 Single-cell RNAseq analysis

The publicly available Seurat object from Nadeu et al. (2022), after quality control, was used for further analysis using the Seurat package in R. he files from the Rendeiro et al. (2020), study were loaded into Seurat using the Read10X() and CreateSeuratObject() functions. Quality control was performed on the second dataset excluding cell barcodes with <1,000 Unique Molecular Identifiers (UMI), <300 detected genes, or mitochondrial expression >15%. In addition, we excluded genes detected in < = 4 cells. Next, we applied in both datasets the functions NormalizeData(), FindVariableFeatures(), ScaleData() and RunPCA() of Seurat (with default parameters). The UMAPs were generated using the function RunUMAP() on the first 30 PCs. To cluster cells into groups, we used the function FindNeighbors (30 PCs) and then determined the clusters based on the function FindClusters().

### 2.4 MYC target genes signature

The signature to score MYC target gene activation was created by integrating data from various sources, including the Hallmark database, Dorothea database, Signature Database from StaudtLab (Schmitz et al., 2018; https://lymphochip.nih.gov/signaturedb/index.html), and ChIP-seq annotated peaks in Burkitt lymphoma (Seitz et al., 2011; Supplementary Figure S1; Supplementary Table S1). To enhance specificity, we excluded genes that overlapped with NOTCH1 due to their shared targeting (Palomero et al., 2006). The MYC target gene signature was scored using ssGSEA method from the GSVA package for bulk analysis and UCell for single-cell resolution. The resulting scores were stratified into high, intermediate, and low activation groups based on quartiles of the score values.

### 2.5 Downstream analysis

Correlation of MYC target gene activation with other pathways, including Hallmark and BCR signaling pathways from KEGG, was performed by scoring all pathways in the same way as MYC target gene activation and calculating the Pearson correlation coefficient. Kaplan-Meier analysis was performed using the survival package in R. Cell annotation was performed manually using the most significant markers in each cluster. Pathway analysis and investigation of cell-cell interactions were conducted using fgsea (Hallmark) and liana in R, respectively. For visualization, we used the Seurat, ggplot2, UpSetR, and ComplexHeatmap packages in R. Chi-square tests were used to test the significance of MYC target gene activation in different genomic alterations. Pearson correlation coefficients were used to correlate two continuous variables.

## 3 Results

We used a comprehensive dataset comprising 263 bulk RNAseq CLL samples from the ICGC cohort without genetic alterations in *MYC* (Knisbacher et al., 2022), along with 12 samples from 6 CLL patients pre- and post-RT (Nadeu et al., 2022). Additionally, we analyzed single-cell RNAseq data from 40,725 cells including CLL, TME and RT (Nadeu et al., 2022). A 285-gene *MYC* target gene signature was created by integrating data from various sources (Methods, Supplementary Figure S1; Supplementary Table S1).

**FIGURE 1:**
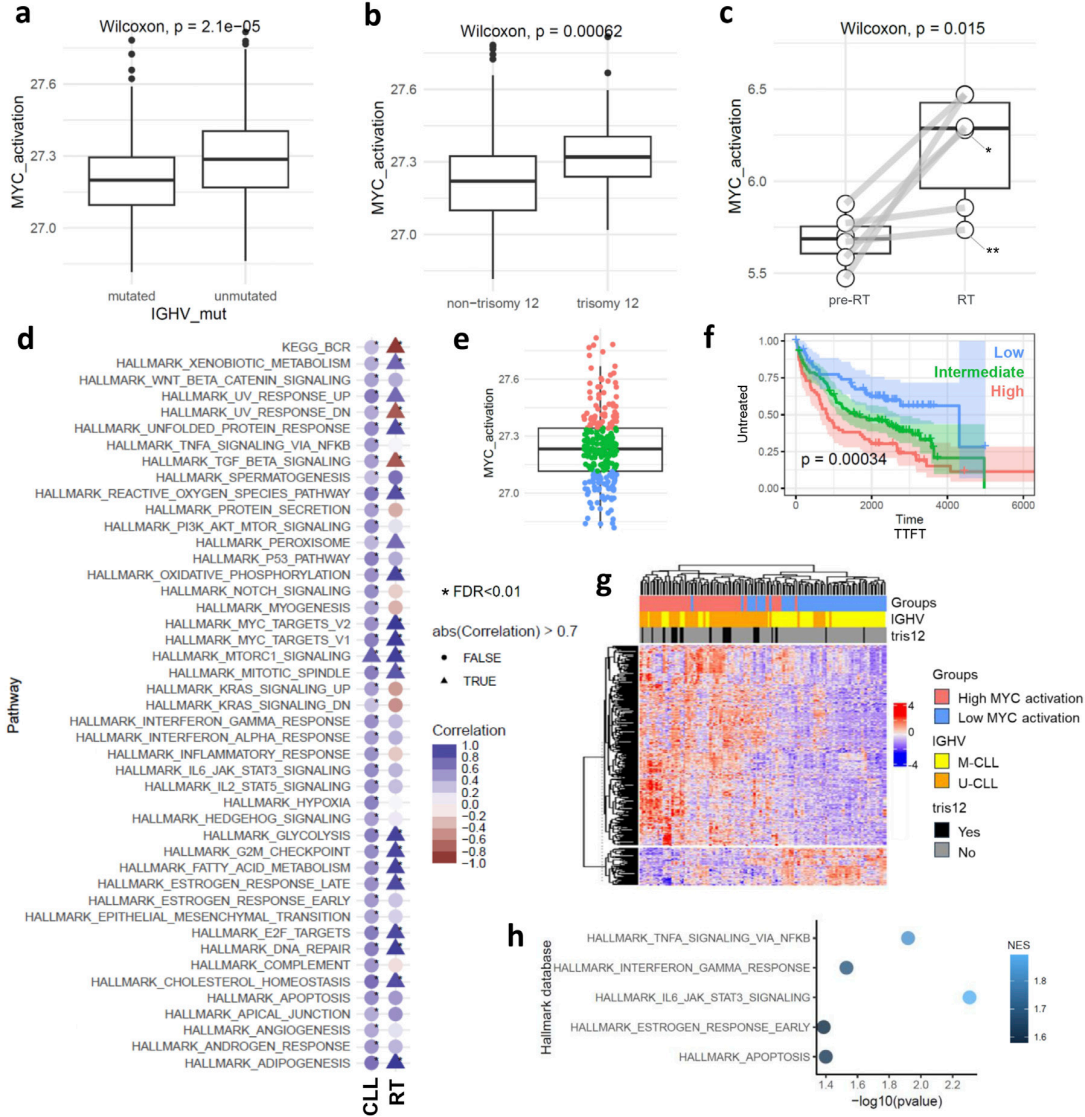
*MYC* target gene activation at bulk level. Boxplots showing *MYC* target gene activation score in: **(a)** mutated and unmutated CLL cases, **(b)** cases carrying or not carrying trisomy 12, **(c)** pre-RT and in RT phase. Asterisks indicate cases with MYC genetic alteration: *translocation, **missense mutation. **(d)** Dot plot displaying the correlation between *MYC* target gene activation scores and the scores of other biological processes in CLL and RT phases. Triangles highlight correlations higher than |0.7| L, while circles represent the others. Asterisks indicate correlations with FDR < 0.01, and the color scale represents the correlation coefficient. **(e)** Boxplot showing the unbiased separation of the CLL cohort into the three subgroups of *MYC* target gene activation. **(f)** Kaplan-Meier curve analysis using TTFT for the high, intermediate, and low subgroups of *MYC* target gene activation. **(g)** Hierarchical clustering is based on the 370 differentially expressed genes between low and high *MYC* target gene activation. **(h)** Pathway analysis using the 370 differentially expressed genes between low and high *MYC* target gene activation.

We initially characterized the activation of *MYC* target genes across clinicobiological subgroups of CLL (Supplementary Figures S2a–c). Our findings show a significant increase in *MYC* target gene activation in U-CLLs compared to mutated IGHV CLLs (M-CLLs) (p < 0.001) (Figure 1a). In terms of gene mutations (Supplementary Figure S2b), cases with HMCN1 mutations showed lower *MYC* target gene activation (Supplementary Figure S2b). Additionally, CLL cases with trisomy 12 exhibited higher levels of *MYC* target gene activation than those without trisomy 12, regardless of IGHV mutational status (p < 0.001) (Figure 1b; Supplementary Figure S2c). Considering the clinical trajectory of CLL, particularly in RT, we observed a statistically significant upregulation of *MYC* target genes in RT compared to pre-RT stages (p = 0.015) (Figure 1c). *MYC* target gene activation showed a strong positive correlation with expression levels of *MYC* in CLL phase (Supplementary Figures S2d,e).

Given the multifaceted role of *MYC* activation, we analyzed its correlation with other pathways to deepen our understanding of its impact in CLL and RT phase (FDR < 0.01 and |r| = 0.3) (Figure 1d). In the CLL phase, *MYC* activation correlated with most of the pathways (45 out of 51 examined), aligning with its known pleiotropic function (Das et al., 2023). Positive correlation was observed between BCR signaling and *MYC* activation in CLL, as expected, validating our methodology (Yeomans et al., 2016). Conversely, in the RT phase, only 19 pathways showed a correlation with *MYC* target gene activation. Notably, a stronger correlation was observed between cell cycling and *MYC* target gene activation in the RT phase, diverging from its broader functional role as seen in CLL. Interestingly, *MYC* target gene activation in RT appeared to be independent of the BCR signaling pathway (negative correlation observed), consistent with literature suggesting a downregulation of this pathway in RT (Nadeu et al., 2022). This lack of association between *MYC* target genes and BCR signaling in RT suggests that MYC may support CLL cell survival via alternative cellular programs, distinct from traditional BCR-mediated mechanisms. Additionally, we noted a loss of correlation between the TGF-beta signaling pathway and *MYC* target gene activation in RT, aligning with previous studies that highlight the diminution of this pathway in RT (Auge et al., 2020).

**FIGURE 2:**
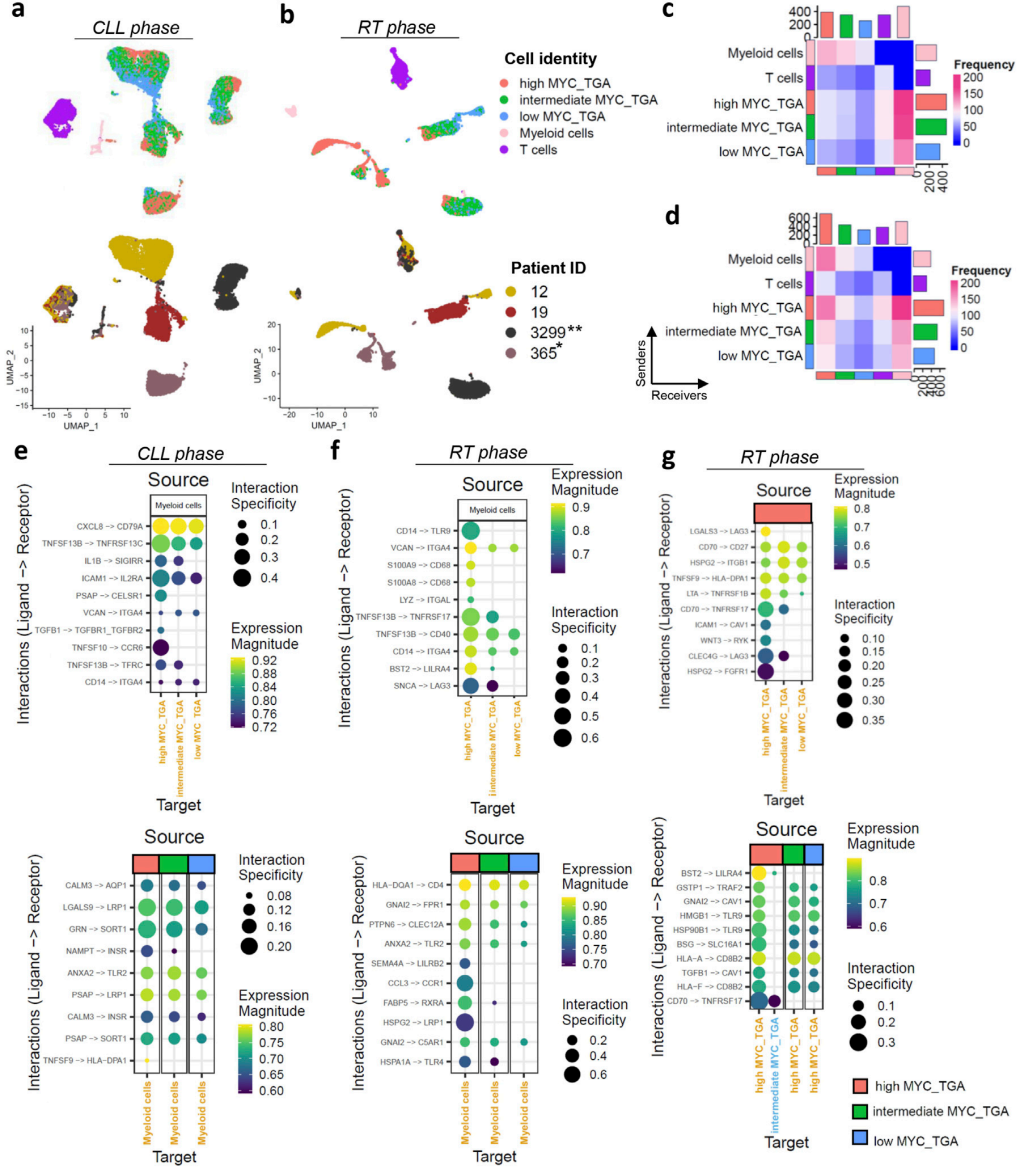
*MYC* target gene activation (MYC_TGA) at single-cell resolution level. **(a,b)** The UMAPs plot displaying the cell identity (malignant cells grouped based on *MYC* target genes activation together with the TME, upper UMAPs) and across the different patients (lower UMAPs) in **(a)** CLL and **(b)** RT phase. The samples were paired in CLL and RT phase derived from the same patients (n = 6) and collected at different time points (T1–T6). Asterisks indicate cases with MYC genetic alteration: *translocation, **missense mutation. **(c,d)** Heatmaps showing the interactions of cells with different cell identities in **(c)** CLL and **(d)** RT phase. The interactions between myeloid and T cells were excluded to focus on the analysis of the malignant cells. **(e–g)** Dot plots display ligand-receptor interactions in **(e)** *MYC* target gene activation categories with myeloid cells in the CLL phase, **(f)** *MYC* target gene activation categories with myeloid cells in the RT phase, and **(g)** interactions within *MYC* target gene activation categories in the RT phase. For panels **(e,f)**, two plots are shown: one with myeloid cells as ligand-expressing cells and the other with myeloid cells as receptor-expressing cells. The size of each dot represents the interaction specificity, while the color indicates the expression magnitude. The identities of the cells were used both as sources and targets.

Next and based on the score of *MYC* target gene activation, we stratified the CLL cases into high, intermediate, and low activation groups based on quartiles of the score values (Figure 1e). Notably, the high activation group includes both M-CLL (n = 40) and U-CLL (n = 49) pre-treatment CLL cases (Supplementary Table S2). Kaplan-Meier curve analysis revealed significant differences among the three *MYC* groups, with high *MYC* target gene activation associated with a significantly shorter time to first treatment (p < 0.001), an effect that remained independent of IGHV mutational status. (Figure 1f; Supplementary Figure S2f). Differential expression analysis between the high and low *MYC* activation groups identified 370 genes (FDR < 0.01) (Figure 1g). Pathway analysis (p < 0.05) linked *MYC* activation to key components of the TME interactions such as ‘*TNFα* signaling via *NFκB*’ (enriched genes: EGR1, IL1B, KLF10, SOCS3, CCL4, CDKN1A, FOS, FOSB, NR4A1, BCL2A1, DUSP4, NR4A3, ICAM1, SGK1, ID2, MYC, GADD45A, MARCKS, FOSL2, PTGS2) and ‘*IL6 JAK STAT3* signaling’ (enriched genes:ITGA4, IL1B, SOCS3, CD38) (Figure 1h). To gain insights into the role of *MYC* target gene activation in TME interactions we examined 27,837 malignant cells and 3,715 cells from the TME (comprising T, and myeloid cells) in CLL phase and 7,479 cells after RT and 1694 TME derived from four samples (Figures 2a,b; Supplementary Figures S3a,b). UMAPs of CLL and RT cells were able to distinguish the TME from malignant cells, however the CLL cell clustering showed high donor specificity (Figures 2a,b).

*MYC* target gene activation represents the principal trajectory of CLL cell orientation across donors, as visualized in the UMAP plots (Figures 2a,b). Notably, in the CLL phase, this trajectory is independent of cell cycle activity (Pearson r = 0.04, Supplementary Figure S3c) highlighting the importance of *MYC* target gene activation in the transcriptomic profiles of the cells. In the RT phase *MYC* activation was higher compared to CLL and the orientation of the cells was influenced by cell cycle as well, further supporting the bulk RNAseq analysis (Pearson R of *MYC* target gene activation and S.Score = 0.39) (Supplementary Figures S3d,e).

High *MYC* target gene activation was associated with increased TME interactions in both CLL and RT phase especially with myeloid cells (Figures 2c,d). There is a notable decrease in interactions from the high to intermediate and low activation groups highlighting the role of *MYC* in the TME communication. Recent studies suggest the *MYC* as a master regulator in host immune response against the cancer but mainly focusing on the mutational activation of *MYC* (Dhanasekaran et al., 2022). The top10 interactions in each comparison showed unique interactions with the TME for high *MYC* target gene activation groups in both CLL and RT phase The RT phase exhibited greater interaction specificity (max = 0.4 in CLL, max = 0.6 in RT) (Figures 2e,f). Interactions with myeloid cells revealed BAFF-R in both phases but different members (*TNFRS13C* in CLL and *TNFRSF17* in RT), with higher interaction specificity in high *MYC* target gene activation. In CLL, *ICAM1*/*IL2RA* and *CXCL8*/*CD79A* interactions showed high specificity for high *MYC* target gene activation (Figure 2e). *CD79A* interactions were not detected in RT, which may be related to the observed downregulation of the BCR signaling pathway *in silico*. RT showed a different set of interactions with microenvironment, notably through *TLR9* (Figure 2f). *TLR9* emerged as a remarkable example of alternative survival pathways post-relapse and RT, promoting CLL cell migration and survival. Considering a recent publication reported high efficacy for combined targeting of *TLR9* and *BTK* (Kennedy et al., 2021), our study suggests that this combination could indeed enhance treatment efficacy and potentially prevent RT. Lower interaction specificity was observed with T cells (Supplementary Figures S3f,g).

Numerous interactions among malignant cells, especially with high *MYC* target gene activation cells, were found, including *BAFF-R*/*CD70*, *CD70*/*CD27*, and *BST2*/*LILRA4* (Figure 2g; Supplementary Figure S3h). Most of these interactions occurred in the RT phase with higher specificity, suggesting an antigen-independent cell-autonomous signaling that leads to *MYC* activation and proliferation, creating a feedback loop that reinforces the malignant phenotype. Our findings, which indicate that higher *MYC* target gene activation is associated with increased interactions with TME, and that high *MYC* levels facilitate communication with other chronic lymphocytic leukemia (CLL) cells, were validated using data from 13,280 cells including both CLL and TME before ibrutinib treatment from the Rendeiro et al. (2020) (Supplementary Figures S4a,b). Furthermore, *MYC* target gene activation was observed to gradually decrease during ibrutinib treatment, with analysis conducted on 10,299 CLL cells before ibrutinib and 9,450 CLL cells during ibrutinib treatment across three different time points (Supplementary Figure S4c). This finding underscores the dependency of *MYC* on BCR signaling in CLL.

## 4 Discussion

Our study highlights the implication of *MYC* in CLL even in the absence of a genetic component. High *MYC* target gene activation was associated with U-CLLs and trisomy 12, treatment outcomes and RT. Additionally, high *MYC* transcriptomic activation showed increased interactions with the TME, suggesting *MYC* as an orchestrator of TME interactions thereby explaining its link to various cellular processes.

In RT, we observed high activation of *MYC* target genes that is independent of the BCR pathway. This activation is highly correlated with cell cycling and may derive survival signals from *TLR9* interactions and malignant-malignant cell interactions. These findings support enhanced patient stratification by considering the transcriptomic activation of *MYC*, especially with new options emerging for targeting the previously “undruggable” *MYC*.

Thus, targeting MYC at the translational level (Largeot et al., 2023) represents an exciting opportunity to complement transcriptomic stratification and may provide therapeutic benefit in MYC-high CLL patients. This could be particularly valuable in the RT setting, where MYC activity is high but uncoupled from canonical BCR signaling. Future studies should investigate whether translational inhibitors could synergize with BTK or TLR9 inhibitors (Largeot et al., 2023), especially in high MYC activation states identified in the present study.

## Data Availability

Publicly available datasets were analyzed in this study. This data can be found here: The bulk RNAseq data from the CLL phase analyzed in this study were obtained from the CLL-map project at https://cllmap.org/downloads.html. The bulk RNA-seq data from RT samples were obtained through kallisto tables, which can be downloaded from https://github.com/ferrannadeu/RichterTransformation/tree/main/bulkRNA-seq/kallisto. The scRNA-seq expression object from the Nadeu et al. study, along with their metadata, is available on Zenodo (https://zenodo.org/records/6631966). Additionally, the single-cell RNA-seq data under ibrutinib treatment were obtained from GEO (GSE111015) as reported by Rendeiro et al. All the scripts for the analysis of bulk and single-cell RNAseq tables up to the visualizations are available (https://github.com/biomedicalGenomicsCNAG/MYCtargetGenes_Activation) with a detailed description of each one.

## References

[B1] Auge H. Notarantonio A. B. Morizot R. Quinquenel A. Fornecker L. M. Hergalant S. (2020). Microenvironment remodeling and subsequent clinical implications in diffuse large B-Cell histologic variant of richter syndrome. Front. Immunol. 11, 594841. 10.3389/fimmu.2020.594841 33381116 PMC7767850

[B2] Das S. K. Lewis B. A. Levens D. (2023). MYC: a complex problem. Trends Cell Biol. 33, 235–246. 10.1016/j.tcb.2022.07.006 35963793 PMC9911561

[B3] Delgado J. Nadeu F. Colomer D. Campo E. (2020). Chronic lymphocytic leukemia: from molecular pathogenesis to novel therapeutic strategies. Haematologica 105, 2205–2217. 10.3324/haematol.2019.236000 33054046 PMC7556519

[B4] Dhanasekaran R. Deutzmann A. Mahauad-Fernandez W. D. Hansen A. S. Gouw A. M. Felsher D. W. (2022). The MYC oncogene - the grand orchestrator of cancer growth and immune evasion. Nat. Rev. Clin. Oncol. 19, 23–36. 10.1038/s41571-021-00549-2 34508258 PMC9083341

[B5] Herishanu Y. Pérez-Galán P. Liu D. Biancotto A. Pittaluga S. Vire B. (2011). The lymph node microenvironment promotes B-cell receptor signaling, NF-kappaB activation, and tumor proliferation in chronic lymphocytic leukemia. Blood 117, 563–574. 10.1182/blood-2010-05-284984 20940416 PMC3031480

[B6] Huh Y. O. Lin K. I. C. Vega F. Schlette E. Yin C. C. Keating M. J. (2008). MYC translocation in chronic lymphocytic leukaemia is associated with increased prolymphocytes and a poor prognosis. Br. J. Haematol. 142, 36–44. 10.1111/j.1365-2141.2008.07152.x 18477041

[B7] Kennedy E. Coulter E. Halliwell E. Profitos-Peleja N. Walsby E. Clark B. (2021). TLR9 expression in chronic lymphocytic leukemia identifies a promigratory subpopulation and novel therapeutic target. Blood 137, 3064–3078. 10.1182/blood.2020005964 33512408 PMC8176769

[B8] Knisbacher B. A. Lin Z. Hahn C. K. Nadeu F. Duran-Ferrer M. Stevenson K. E. (2022). Molecular map of chronic lymphocytic leukemia and its impact on outcome. Nat. Genet. 54, 1664–1674. 10.1038/s41588-022-01140-w 35927489 PMC10084830

[B9] Largeot A. Klapp V. Viry E. Gonder S. Fernandez Botana I. Blomme A. (2023). Inhibition of MYC translation through targeting of the newly identified PHB-eIF4F complex as a therapeutic strategy in CLL. Blood Adv. 7, 3619–3637. 10.1182/blood.2022017839 PMC10646824 37084385

[B10] Nadeu F. Royo R. Massoni-Badosa R. Playa-Albinyana H. Garcia-Torre B. Duran-Ferrer M. (2022). Detection of early seeding of richter transformation in chronic lymphocytic leukemia. Nat. Med. 28, 1662–1671. 10.1038/s41591-022-01927-8 35953718 PMC9388377

[B11] Palomero T. Lim W. K. Odom D. T. Sulis M. L. Real P. J. Margolin A. (2006). NOTCH1 directly regulates c-MYC and activates a feed-forward-loop transcriptional network promoting leukemic cell growth. Proc. Natl. Acad. Sci. U. S. A. 103, 18261–18266. 10.1073/pnas.0606108103 17114293 PMC1838740

[B12] Rendeiro A. F. Krausgruber T. Fortelny N. Zhao F. Penz T. Farlik M. (2020). Chromatin mapping and single-cell immune profiling define the temporal dynamics of ibrutinib response in CLL. Nat. Commun. 11, 577. 10.1038/s41467-019-14081-6 31996669 PMC6989523

[B13] Schmitz R. Wright G. W. Huang D. W. Johnson C. A. Phelan J. D. Wang J. Q. (2018). Genetics and pathogenesis of diffuse large B-Cell lymphoma. N. Engl. J. Med. 378, 1396–1407. 10.1056/NEJMoa1801445 29641966 PMC6010183

[B14] Seitz V. Butzhammer P. Hirsch B. Hecht J. Gütgemann I. Ehlers A. (2011). Deep sequencing of MYC DNA-Binding sites in burkitt lymphoma. PLoS One 6, e26837. 10.1371/journal.pone.0026837 22102868 PMC3213110

[B15] Wang Y. Ding W. (2020). Richter transformation of chronic lymphocytic leukemia in the era of novel agents. Clin. Adv. Hematol. Oncol. 18, 348–357. 32649656

[B16] Yeomans A. Thirdborough S. M. Valle-Argos B. Linley A. Krysov S. Hidalgo M. S. (2016). Engagement of the B-cell receptor of chronic lymphocytic leukemia cells drives global and MYC-Specific mRNA translation. Blood 127, 449–457. 10.1182/blood-2015-07-660969 26491071

