# Supplementary material for: MYC target gene activation in chronic lymphocytic leukemia and richter transformation: links to aggressiveness and tumor microenvironment interactions
Source: Front Pharmacol. 2025 Aug 15;16:1642458. doi: 10.3389/fphar.2025.1642458 (PMC12394460; doi:10.3389/fphar.2025.1642458)
Supplement: Supplementary file 1 [file DataSheet1.pdf]

Supplementary Material

Supplementary Table 1. The reported MYC target gene signature.

MYC target gene signature					
ABCB9	CUX1	HSP90AB1	NOLC1	RPL19	TOMM20
ABCC4	CYBA	HSPA8	NOP56	RPL32	TP73
ACOT7	DDIT4	HSPA9	NPM1	RPL5	TPP2
ACP1	DDX1	HSPD1	NR1D1	RPL6	TRA2B
ACYP2	DDX10	HSPE1	NRAS	RPLP0	TRAF2
AGPS	DDX18	HSPH1	NRP2	RPS19	TRAP1
AIMP2	DGKD	IARS	NUFIP1	RPS2	TRAPPC9
AKAP1	DHX15	ICAM1	NUP153	RPS20	TRIP13
ANKMY1	DNAJB6	IDH3B	NUP155	RPS3	TTC7A
ANKRD11	DPYSL2	IER2	ODC1	RPS5	TXNL4A
ANP32B	EHMT1	IFI30	OSBPL3	RPS6	TYMS
APBB2	EIF2S1	IFRD2	PA2G4	RPS6KA5	U2AF1
APEX1	EIF3B	IMPDH2	PAICS	RPTOR	UBA52
ARF4	EIF3J	INPP5A	PARP1	RRP9	UBE2C
ARFGAP2	EIF4A1	IPO7	PCBP1	RUVBL2	UBE2D3
ARID1A	EIF4H	IQSEC1	PER1	SCARB1	UBE2E1
ARID3A	EIF5	KARS	PFKM	SEC14L1	UBXN8
ARPC5L	EML2	KDM4B	PFN1	SET	UMODL1
ARSB	ENO1	KLHDC4	PHB	SF3A1	UNG
ATF4	EPB41	LBR	PHB2	SGMS1	USP1
ATF7	EPS15L1	LDHA	PLEC	SIGMAR1	UXT
ATXN10	ETF1	LMNA	PMM2	SLC19A1	VAV2
BCL11A	ETFA	LRRK1	PNPLA7	SLC25A3	VDAC1
BMI1	ETS2	LTA4H	POLD2	SLC39A11	VOPP1
BMP7	FADS2	LTBP4	POLE3	SLC3A2	XPO1
BUB3	FAH	LZTR1	PPARGC1B	SLC6A6	XRCC6
C1QBP	FASN	MAD1L1	PPIA	SLC9A1	YBX1
CAD	FBL	MAN2A1	PPM1G	SMAD6	YWHAE

Supplementary Material

CARM1	FKBP2	MAPK1	PRCC	SMARCA 2	YWHA Q
CBS	FKBP5	MCM3	PRDM15	SMARCA D1	ZBTB7B
CBX3	FNDC3B	MCM4	PRDX3	SND1	ZFAT
CCDC6	FOXK1	MEN1	PRKCE	SNRPA	ZFP36
CCDC88 C	FOXN3	METTL1	PSMC4	SNRPB2	ZFP36L 2
CCNA2	FOXP1	MFNG	PSMD14	SNRPD1	ZMIZ1
CCND3	FPGS	MNT	PSMD3	SNRPD3	ZNF274
CCT5	GALNT2	MPHOSP H6	PSMD7	SOX12	
CCT7	GNL3	MRPL23	PSME3	SRM	
CD164	GSPT1	MRPS18B	PSMG1	SRPK1	
CD63	HDAC2	MSH2	PTGES3	SSB	
CDC25B	HDGF	MTHFD1L	PTMA	STAT3	
CDCA7	HERPUD1	MTSS1	PWP1	SYK	
CDK4	HMGN2	MYCBP2	PYCR1	SYNCRIP	
CDKN2 A	HNRNPA1	MYH9	RAI1	TBCD	
CHCHD 6	HNRNPA2 B1	NAP1L1	RALGDS	TBL1XR1	
CLEC16 A	HNRNPA3	NAT10	RANBP3	TFAM	
CLNS1A	HNRNPD	NCBP1	RBM15B	TFAP4	
CSK	HNRNPF	NCBP2	RBM39	TFEB	
CSTF3	HNRNPL	NCOR2	RFX3	TKT	
CTSC	HNRNPR	NHP2	RPL13	TLE3	
CUL1	HNRNPU	NME1	RPL18	TMED10	

Supplementary Table 2. Number of M- and U- CLL cases in the 3 MYC target gene activation cases

MYC target gene activation	M-CLL	U-CLL
High	24	42
Intermediate	92	36
Low	47	19

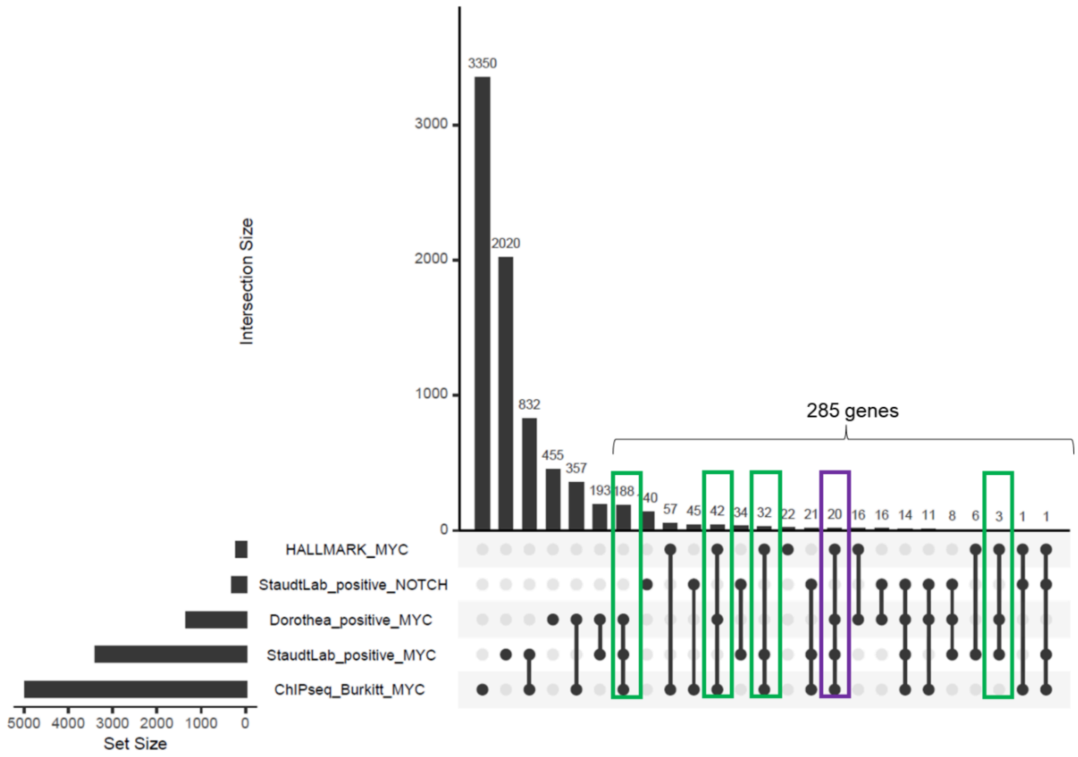

Supplementary Figure 1. A 285-gene *MYC* target gene signature. The signature to score *MYC* target gene activation was created by integrating data from various sources, including databases and experimental B cell malignancy datasets. Genes that overlapped with NOTCH1 were excluded from the analysis. The green boxes highlight the intersections of genes present in 3 out of the 4 sources, while the purple boxes highlight the intersections of genes present in all 4 sources. The bar chart displays the intersection size, indicating the number of genes shared among the different datasets.

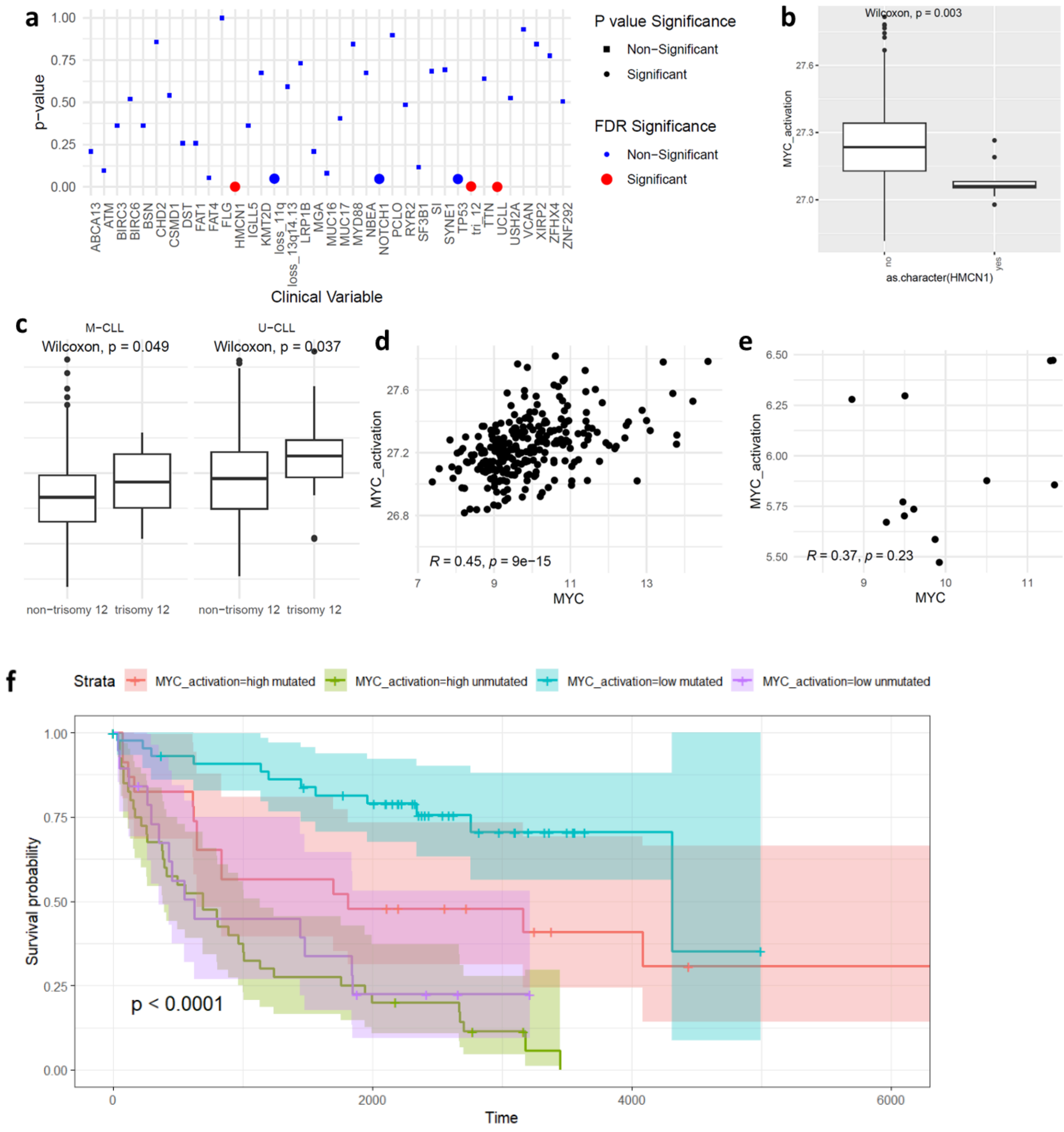

Supplementary Figure 2. Analysis of *MYC* target gene activation at bulk level. a Dot plot displaying the p-values of various clinical variables associated with *MYC* target gene activation. Variables with significant p-values are displayed in circles, and those with significant FDR are marked in red. b Box plot comparing *MYC* target gene activation levels based on the presence of *HMCN1* mutation. c Box plots showing *MYC* target gene activation in non-trisomy 12 versus trisomy 12 cases in M-CLL and U-CLL cases. d Scatter plot illustrating the correlation between *MYC* expression and *MYC* target gene activation in CLL. e Scatter plot illustrating the correlation between *MYC* expression and *MYC* target gene activation in RT. f Kaplan-Meier survival curve comparing high and low *MYC* target gene activation groups within IGHV mutational status categories.

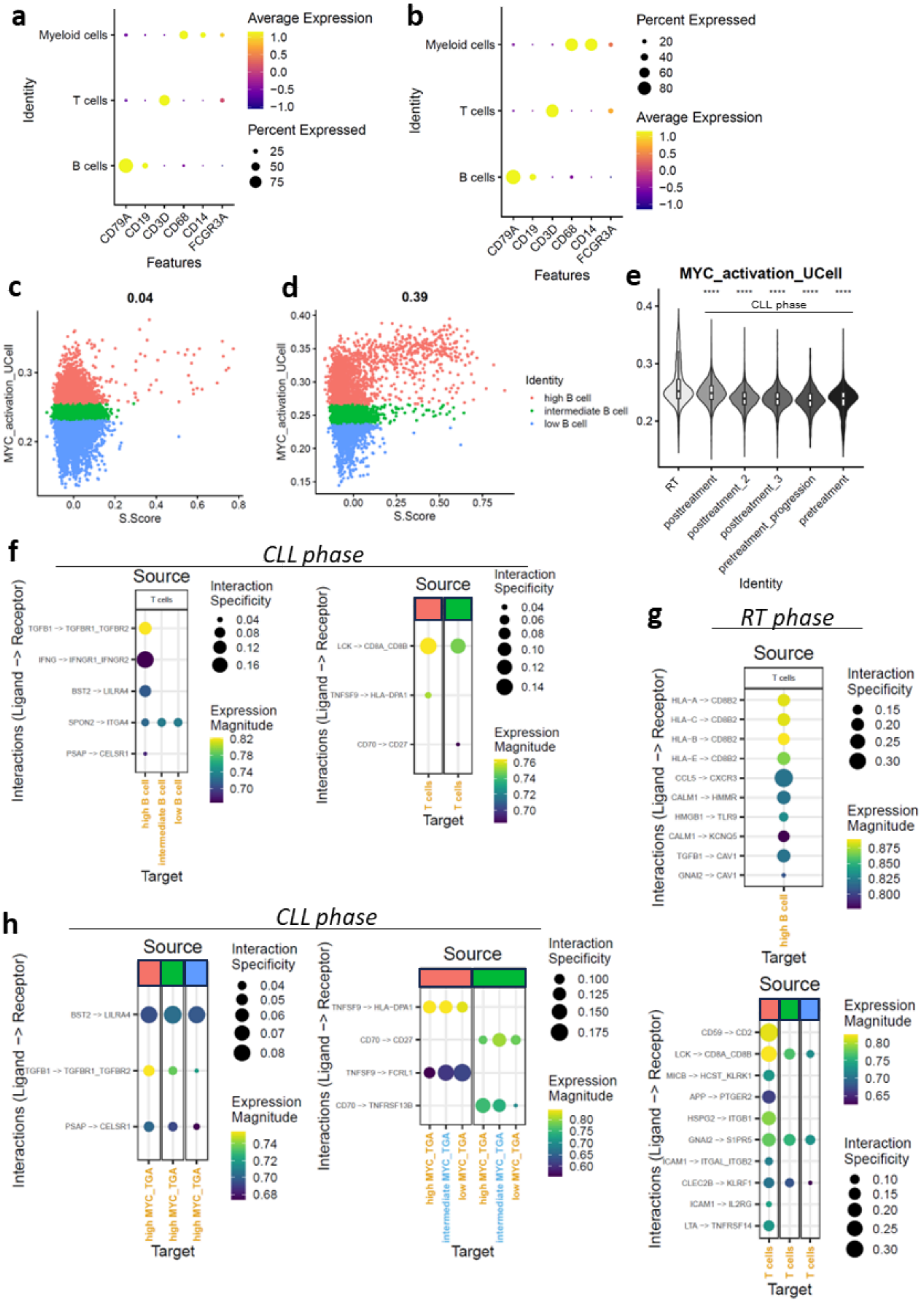

Supplementary Figure 3. Analysis of *MYC* target gene activation with single-cell resolution. a Dot plot showing the average expression and percentage of expression of specific marker genes in

myeloid cells, T cells, and B cells in CLL phase. b Dot plot displaying the average expression and percentage of expression of specific marker genes in myeloid cells, T cells, and B cells in RT phase. c-d Scatter plot illustrating the correlation between *MYC* target gene activation and S.Score in c CLL cells and d RT cells. Cells are color-coded based on B cell identity: high B cell, intermediate B cell, and low B cell. e Violin plot comparing *MYC* target gene activation across different CLL phases and conditions. Asterisks displaying the statistically significant differences ($p<0.001$) for each CLL timepoint compared to RT phase. f-h Dot plots displaying ligand-receptor interactions between different cell identities within *MYC* target gene activation categories. The size of each dot represents the interaction specificity, while the colour indicates the expression magnitude. Panels illustrate interactions of: f. *MYC* target gene activation categories cells with T cells in CLL phase, g. *MYC* target gene activation categories cells with T cells in RT phase and h. *MYC* target gene activation categories in the CLL phase.

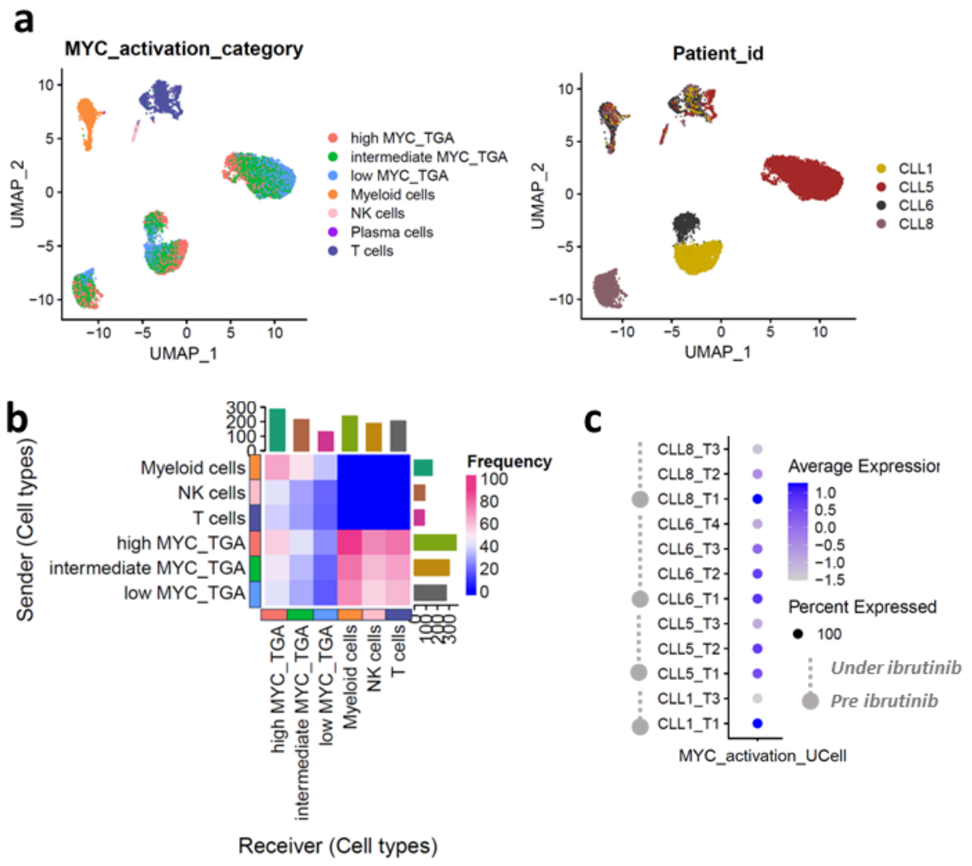

Supplementary Figure 4. Single-cell analysis of *MYC* target gene activation before and during ibrutinib treatment. a UMAP plot displaying the cell identities based on *MYC* target gene activation categories (left) and the distribution of cells across different patients (right) at the first time point before ibrutinib treatment. b Heatmap showing the frequency of interactions between TME and *MYC* activation categories at the first time point before ibrutinib treatment. The interactions between myeloid, T, and NK cells were excluded to focus on the analysis of the malignant cells. c Dot plot

displaying the average expression and percentage of expression of *MYC* target gene activation across different time points and conditions (pre-ibrutinib and under ibrutinib treatment) across patients. The size of each dot represents the percentage of cells expressing *MYC* activation, while the colour indicates the average expression level.
